# Development of a Modular and Equitable Surgical Simulator

**DOI:** 10.9745/GHSP-D-21-00744

**Published:** 2022-06-29

**Authors:** Yihan Lin, Jason J. Han, John J. Kelly, Anna K. Gergen, Emily Downs

**Affiliations:** a Division of Cardiothoracic Surgery, Department of Surgery, University of Colorado, Aurora, CO, USA.; b Division of Cardiovascular Surgery, Department of Surgery, University of Pennsylvania, Philadelphia, PA, USA.

## Abstract

There is a tremendous need for affordable and accessible surgical simulators in the United States and abroad. Our group developed a portable, modular, inexpensive surgical simulator designed for all levels of surgical trainees, from medical students to cardiothoracic surgery fellows, and adaptable to a variety of surgical specialties. Our goal is to provide a platform for innovative surgery simulation that applies to any learner or resource setting. We describe the development, assembly, and future directions for this simulator.

## Abstract (precis)

Current trends in surgical simulation favor high-fidelity, costly models that are often limited to high-income academic centers. The GlobalSurgBox overcomes many of the barriers to routine implementation and use of surgical simulators in low-income countries by circumventing the often prohibitive financial, time, and personnel investments required of current simulation prototypes.

## Key Messages

- Simulation training is a critical component of surgical education.
- Despite its potential importance in advancing the field of surgical training, successful implementation of surgical simulation models is difficult in low-resource settings due to limitations in affordability and accessibility.
- The GlobalSurgBox is a portable surgical trainer adaptable to all training levels, surgical specialties, and resource settings, overcoming many of the existing barriers to the implementation and use of current surgical simulation models.

## INTRODUCTION

The importance and efficacy of simulation in surgical training are widely acknowledged globally and in low- and middle-income countries (LMICs).^1^^–^^3^ However, current training platforms are limited by affordability, portability, accessibility, and ease of implementation into existing training programs as well as individual routines. And, compared to the United States, surgical training opportunities in low-resource environments are often far more limited.

The paradigm of low-fidelity simulation models has gained recent momentum as a strategy to overcome the aforementioned barriers, especially in low-resource settings. While high-fidelity systems are traditionally thought to provide a more realistic representation of surgical skills, implementation of low-cost surgical simulation models and training programs have demonstrated reasonable success following application in a variety of LMICs.^4^^–^^8^ Among these include a low-fidelity ear surgery simulator for acquisition of otolaryngology skills and a penile model to teach male circumcision, both of which have been validated as cost efficient and useful simulation models at Uganda-based training facilities.^9^^,^^10^ Similarly, a low-cost model simulating an open inguinal hernia repair has been validated as a useful tool for surgical trainees.^11^ Importantly, accruing data suggest that skill acquisition from low-fidelity simulators is noninferior to those gained from high-fidelity models.^12^^–^^14^ A literature review by Lefor et al. demonstrated that in 15 of 17 included studies, procedure skill was equivalent after training with low-fidelity versus high-fidelity simulators.^14^ Similarly, random allocation of participants to either a high- or low-fidelity simulation training session resulted in equal or even worse performance and growth in knowledge among the high-fidelity simulation users compared to the low-fidelity simulation users.

Low-fidelity simulators enable trainees to practice surgical skills regardless of their location, academic affiliation, time constraints, or resource stratum, which has potentially powerful implications for global health and medical education.^15^^–^^17^ We designed a low-cost, portable surgical simulator to help overcome resource constraints as well as address many of the commonly reported barriers to the routine use of currently available high-fidelity simulators, allowing for easy and affordable implementation. We describe this platform and its potential for developing a sustainable global surgical education initiative, inviting ongoing innovation for trainees to create future iterations based on local needs.

Low-fidelity simulators enable trainees to practice surgical skills regardless of their location, academic affiliation, time constraints, or resource stratum.

## GLOBALSURGBOX SURGICAL TRAINER

**FIGURE 1:**
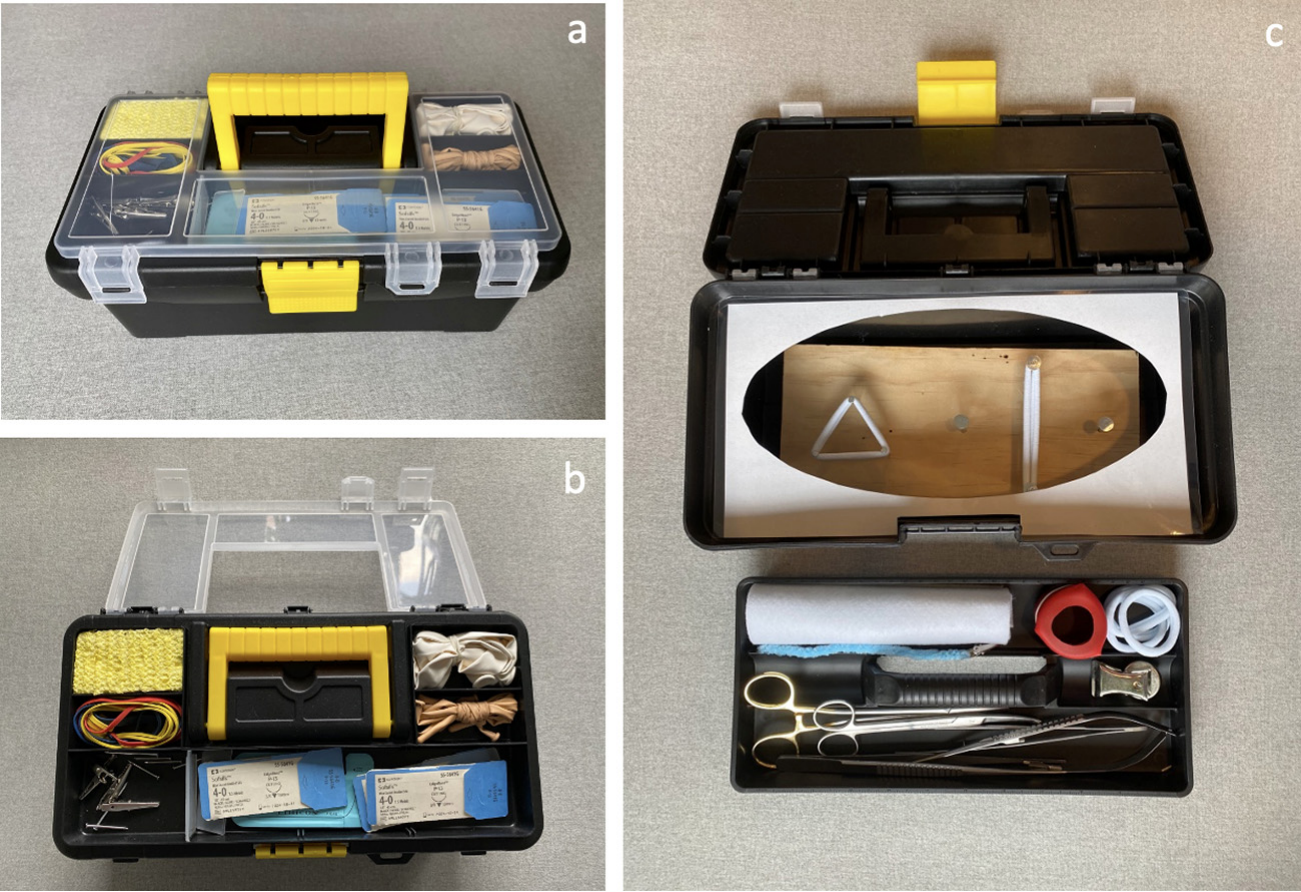
GlobalSurgBox Components (a) The GlobalSurgBox is a comprehensive surgical trainer that fits within a toolbox. (**b)** The lid holds all the necessary materials for building and performing the training modules. (**c)** A removable tray holds the necessary surgical instruments, including several different types of needle drivers, forceps, and scissors, while a removable wooden board located at the base of the toolbox serves as the foundation for individual training exercises.

The GlobalSurgBox is a surgical trainer that is housed entirely within a 12.5-inch toolbox. The lid of the toolbox has built-in compartments to store sutures, nails, and other supplies used for the simulator modules. Inside the toolbox, a removable tray contains the necessary surgical instruments including needle drivers, forceps, and scissors. At the base of the toolbox, a wooden board serves as the foundation for all surgical training exercises (Figure 1).

A complete manual detailing how to make the GlobalSurgBox and implement this simulator on both a local and global scale is provided in a Supplement. In the United States, the GlobalSurgBox costs approximately US$25 to create. While materials to construct the simulator are readily available in the United States, we recognize these exact items may be more challenging to find in resource-limited countries. One of the benefits of the GlobalSurgBox is that it can be modified to suit any resource setting, with the intent that materials are sourced from home or locally and with appropriate substitutions.

The Table provides an example of locally accessible materials and the associated cost breakdown to build 1 GlobalSurgBox in Rwanda, which totals less than US$10 (Table). The majority of commercially available simulators utilize materials that are expensive and difficult to replenish, such as animal tissue or silicone. Our model prioritizes the use of universally available materials such as cotton, rubber, and wood, all of which can be purchased in bulk to keep costs low. Surgical instruments and suture remain the most challenging and expensive items to find, and thus we rely heavily on donations to supply these materials, particularly in resource-limited countries. When available, unused suture and rejected surgical instruments unsuitable for patient care may be acquired from operating room staff and repurposed for the simulator.

**TABLE.** Cost of Materials to Create 1 GlobalSurgBox

	Price, RWF^a^
Hair ties (5)	1,500
Thread	50
Sport mat	280
Shoelaces	150
Balloons (7)	1400
Rubber bands (5)	500
Pen	500
Ruler	500
Wood and nails	3000
Alligator clips	1500
Sponge	150
Leather	150
Toilet paper roll	150
Total	9,830^a^

Abbreviation: RWF, Rwandan franc.

a Equal to less than US$10.

## TRAINING MODULES

The GlobalSurgBox was designed as a global surgical initiative that encourages learners to adapt the apparatus to address their individualized training needs. The core design is modular, allowing trainees to easily change and redesign exercises. Medical students, general surgery residents, and subspecialty surgical fellows at the same institution can share a single GlobalSurgBox to create separate, individualized curricula. Using intentionally placed nails and other materials, an infinite number of exercises can be designed to fit the needs and skills of the specific user.

The GlobalSurgBox's core design is modular, allowing trainees to easily change and redesign exercises.

**FIGURE 2:**
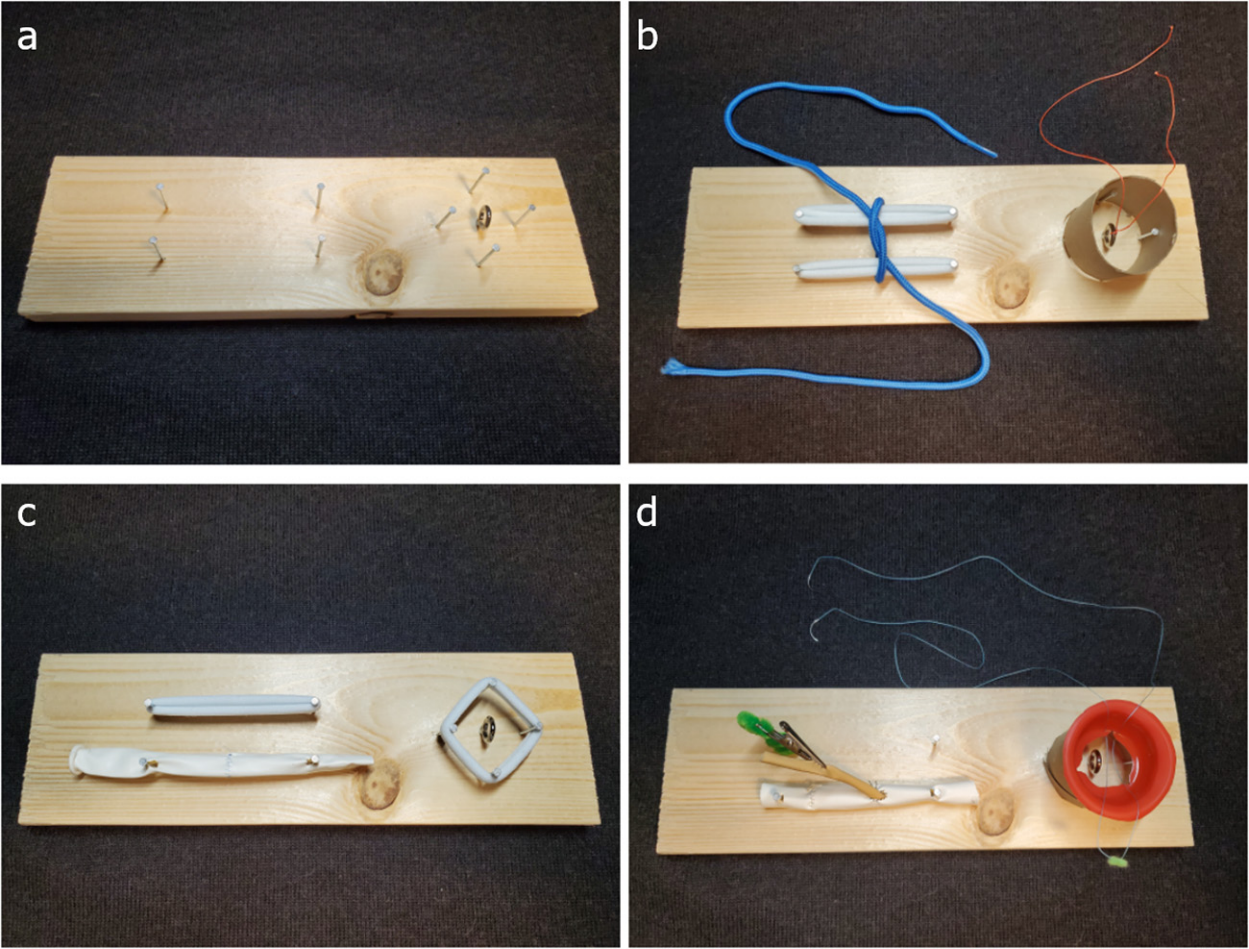
Sample Modules **(a)** The base of the simulator includes a wooden board (11 in x 3.5 in), nails (1.5 in), and an eye hook screw. This serves as the starting platform from which all modules are built using additional materials stored within the toolbox. **(b)** Medical student module #1 shows tying square knots with hair ties and a shoelace; #2 shows tying in a hole using a toilet paper roll and fishing line. **(c)** General surgery module #1 shows end-to-end vascular anastomosis using a linear balloon; #2 shows needle angle practice using hair ties. (**d)** Cardiothoracic surgery resident/fellow module #1 shows end-to-side coronary anastomosis using pipe-cleaners, alligator clip, and linear balloons; #2 shows aortic valve module using a cut cupcake holder placed inside a toilet paper roll.

We created modules appropriate for all levels of training (Figure 2).

- Medical student:
  - Two-handed square knots (Figure 2b)
  - One-handed square knots
  - Basic suturing (Figure 2c)
- Surgery resident:
  - Needle angle practice
  - End-to-side bowel anastomosis
  - End-to-end vascular anastomosis
  - Vascular patch anastomosis (Figure 2d)
- Cardiac surgery fellow:
  - End-to-side vascular anastomosis (proximal CABG)
  - End-to-side vascular anastomosis (distal CABG)
  - Aortic valve module

**FIGURE 3:**
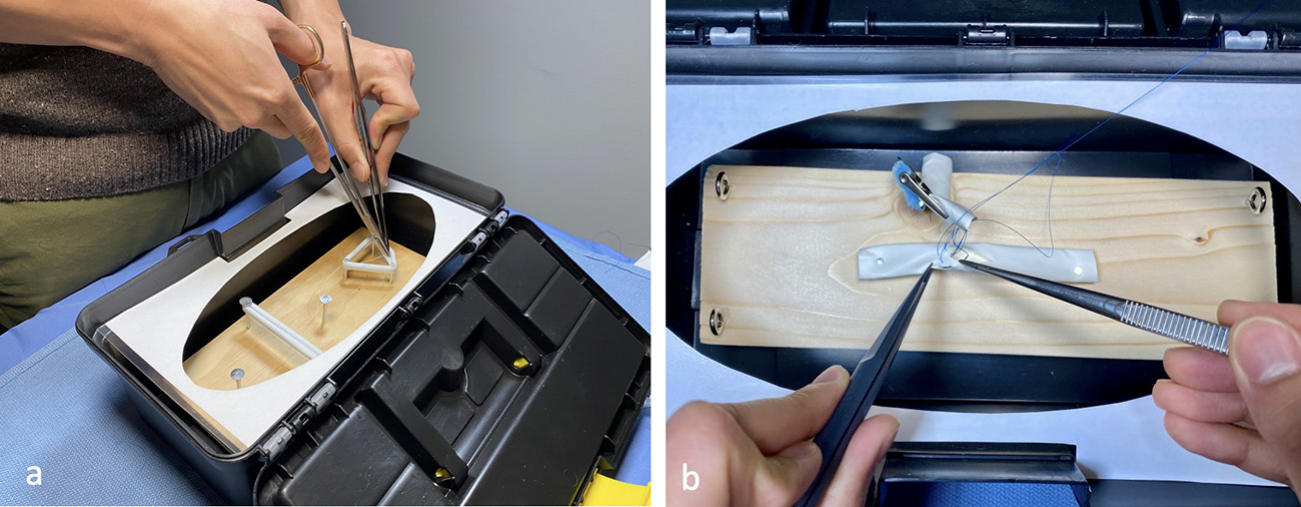
Practicing With the GlobalSurgBox **(a)** Suturing module: Nails are positioned on the wooden board in linear/triangular shapes. Hair ties are wrapped around the nails to simulate tissue. This allows the practice of forehand, backhand, and mattress suturing. These drills can be practiced with a full-length suture to learn suture spacing or a needle with only a small suture tail to hone needle angles and forceps agility. **(b)** End-to-side coronary/vascular anastomosis module: A balloon is stretched across 2 nails attached to the wooden board to simulate the target vessel, while a second balloon suspended by pipe cleaners and an alligator clip serves as the graft. Using a polypropylene suture, the anastomosis is started. The alligator clip can be tilted downward to simulate “parachuting” of the graft to the target vessel.

These modules were designed with the intent to reinforce fundamental operative skills used throughout one's surgical career. All of the modules can be executed either with the wooden board positioned outside the toolbox to replicate suturing close to the skin level or inside the toolbox to simulate the depth and needle angles of a sternotomy, thoracotomy, or laparotomy (Figure 3).

## LESSONS LEARNED

The following quotes are a few examples of the sage advice that has inspired surgical trainees to innovate, practice, and use what is readily available to them to develop the technical skills necessary for surgery.^18^

*Practice passing a 7-0 suture through a bar of soap and tie it down without pulling it through…*

*Boil noodles and sew them together without tearing…*

*Tie a silk suture on the tab of an empty soda can without lifting the can off the table …*

We foresee tremendous potential in the use of multipurpose, low-fidelity simulators, such as the GlobalSurgBox.

1. It can be used at home and easily transported to and from the hospital depending on the trainee's situation and schedule.
2. Trainees can perform exercises in limited timeframes, such as while a patient is being prepared for surgery by the anesthesia team.
3. The simulator can be used as an effective teaching tool for senior team members to demonstrate surgical maneuvers to medical students or junior trainees.
4. The durability and portability of a toolbox overcome many of the challenges related to setting up and using a traditional simulation lab.

## FUTURE DIRECTIONS

The GlobalSurgBox kits are intended to be made locally to enable easy adoption and incorporation of simulators into local training programs. While the ultimate goal is for each trainee to own a personal GlobalSurgBox to allow them to practice skills in any setting, this may not be realistic during the early phases of implementation. One kit may therefore serve multiple trainees provided the simulator remains stationed in a shared workspace. Resource guides and instructional videos are also available on our website (globalsurgbox.com) to ensure learners use proper technique, even when practicing independently.

While the initial construction of the GlobalSurgBox was created and informed through consulting with colleagues from LMICs, we anticipate forming additional global partnerships during the distribution phase of the simulator to mutually guide the direction of our collective endeavor and ensure alignment with local needs.

As an example of how to scale this project, we have committed to implementing a “make one locally, give one globally” model through which for each box made locally, a second box is made and donated to our global partners in LMICs. We challenge all participants with additional resources to adopt this model.

Before widespread implementation, however, it is imperative to validate the usefulness of the simulator in real-world settings, including investigation at both United States-based surgery training programs and training programs in LMICs.^19^ After distribution of the simulator and in-person coaching on how to perform a variety of common surgical maneuvers, trainees should be allowed to practice ad libitum for a specified period. The simulator's usefulness and feasibility can then be assessed using qualitative and quantitative methods, including anonymized participant surveys and timed completion of modules.

The outlined approach for creating the simulator and designing associated training modules enables a blank slate for deliberate practice. We hope for additional future applications of the simulator devised by trainees around the world, with the goal that the GlobalSurgBox will serve as a sustainable method of promoting equity and advancement in the field of surgical education on a global scale.

## Supplementary Material

GHSP-D-21-00744-supplement.pdf

## Notes

**Peer Reviewed**

**First published online:** June 6, 2022.
