# Supplementary material for: Development of a Modular and Equitable Surgical Simulator
Source: Glob Health Sci Pract. 2022 Jun 29;10(3):e2100744. doi: 10.9745/GHSP-D-21-00744 (PMC9242608; doi:10.9745/GHSP-D-21-00744)
Supplement: GHSP-D-21-00744-supplement.pdf [file GHSP-D-21-00744-supplement.pdf]

Supplement to: Lin Y, Han JJ, Kelly JJ, Gergen AK, Downs E. Development of a modular and equitable surgical simulator. Glob Health Sci Pract. 2022;10(3):e2100744. https://doi.org/10.9745/GHSP-D-21-00744



# How to Make your own GlobalSurgBox

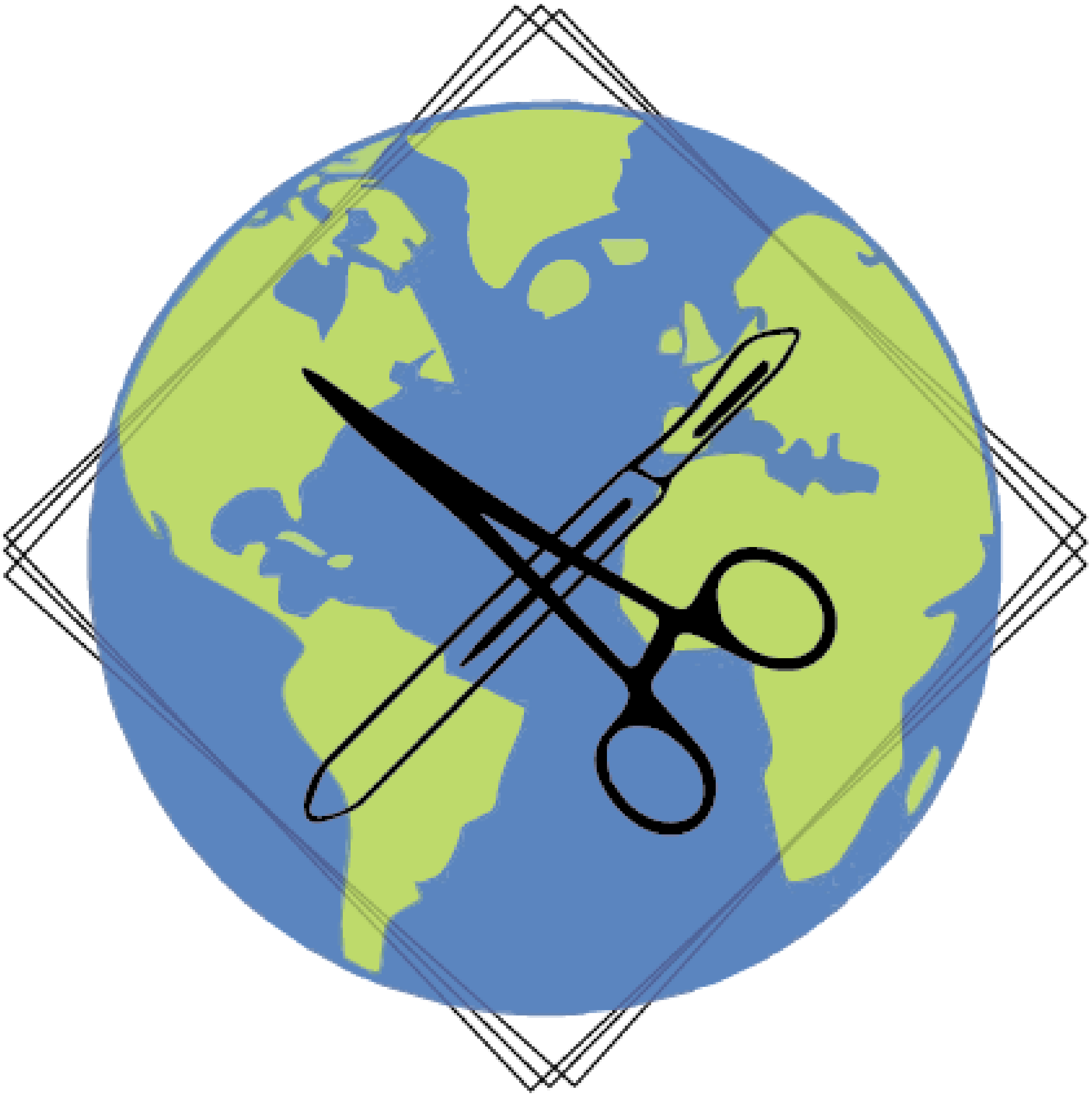

# Table Of Contents

**About this Project** 3

**How to Apply for Funding** 4

**Gathering Supplies** 6

**Box Building Tips** 7

**Skills Night** 7

**GlobalSurgBox Setup Instructions** 8

- Appendix 1: Real-size templates for wooden boards 12
- Appendix 2: Laminated Name Tags 13
- Appendix 3: Description Cards 14
- Appendix 4: Skills Night Poster 15
- Appendix 5: Request for Surgical Supplies 16

# About this Project

**Mission of the GlobalSurgBox**

The vision of the GlobalSurgBox is to make surgical simulation and its practice accessible to learners of all training levels. We designed a portable surgical simulator that fits completely within a 12 inch toolbox, with materials that are affordable, reusable, and easily sourced. This simulator allows for innovation by any of its users, whether it be a medical student, surgery resident, or advanced surgical fellow. Faculty will also find this very useful for teaching purposes. Additionally, the GlobalSurgBox can be modified to suit any resource setting, with materials sourced locally to ensure continued practice. Finally, video training modules are being updated on the GlobalSurgBox website (www.globalsurgbox.com) regularly to aid in the development of surgical skills.

**Global Health Equity**

This simulator was designed with global health equity as its core mission. We want to make surgical training accessible to anyone around the world, including our colleagues in low and middle income countries (LMICs). We encourage every GlobalSurgBox user to consider health equity and improve access to simulation every time you make or use the GlobalSurgBox. See our “make one, give one” model below.

**The Goal of this Manual**

This manual seeks to enable learners to build the GlobalSurgBox at scale. We hope this will increase the ability for distribution to LMICs and increase the number of learners that are reached.

**The University of Colorado Global Surgery Student Alliance (CU GSSA)**

We challenge all users of this document with additional resources to adopt a “make one locally, give one globally” model. As an example of how to scale this project, our group, the CU GSSA, have committed to a model by which for each box made locally, a second box is made and donated to our global partners. We have used this document to make 50 boxes at a time (1-2 times per year), with 25 to be used by our local students, and 25 boxes that we send to partners in LMICs. Therefore this manual outlines how to apply for funding, bring together GSSA executive members to build the 50 boxes, host an event to teach 25 of our local medical students how to use the box (and then send them home with one), and finally send the remainder boxes to a partner in an LMIC.

**For further information**

This document was created by the University of Colorado Global Surgery Student Alliance with mentorship from Dr. Yihan Lin. For questions or suggestions, please email globalsurgbox@gmail.com, cugssa@gmail.com and yihan.lin@gmail.com.

This manual was last updated on 2022.03.30.

# How to Apply for Funding

1. Why is funding important?

- Available surgical simulators on the market are often expensive, making them inaccessible to all learners. The GlobalSurgBox attempts to circumvent this barrier by developing a kit for ~ $25 per box. This cost will vary based on donations received and modules you wish to include, as this will modify the surgical instruments required in the box. Securing grant or scholarship funding helps to alleviate the financial burden on learners, especially students and those in LMICs. Furthermore, securing funding allows for scalability of the project.

2. What sorts of funding mechanisms are available?

- Institutional grants/scholarships
  - Example of successful grants at the University of Colorado: https://coloradosph.cuanschutz.edu/research-and-practice/centers-programs/globalhealth/education/scholarships
- National/international grants/scholarships
  - Example: https://www.aafp.org/family-physician/patient-care/global-health/education/scholarships-funding.html
- Individuals (e.g. faculty) invested in surgical education and/or global health

3. How to increase chances of success for a grant application

- Most applications will ask for a project plan, budget, personal statement, and letters of recommendation. Start early, these applications can take weeks to complete. Here, we (the CU GSSA) share a sample funding application which we submitted, which you are free to use and modify for your own funding efforts.

**Project Plan Example:**

As the development of new surgical technologies and techniques rapidly increase, ongoing training is essential now more than ever. Surgical simulation fills an ever-expanding role in such original and continuous education, both in the US and abroad. In high-income countries (HICs), this learning frequently occurs in state-of-the-art facilities under the guidance of dedicated mentors and advanced surgical simulators. Simulators currently used include high-cost, high-fidelity computer-based training, synthetic figures, and organic models, such as anesthetized animals and human cadavers. Institutions in many HICs can financially support such educational measures and have access to supporting systems, such that learners generally have access to various forms of simulator training and online resources. However, this stands in contrast to the training options available in many low- and middle-income countries (LMICs), wherein various economic, political, cultural, medical, and systemic barriers prevent access to simulators. Over 16 million lives were lost in 2010 due to surgical conditions and, in low- and middle- income countries, 1.5 million deaths were avertable if basic surgical care was widely accessible. A significant component of positive operative outcomes and patient safety relates directly to experience in initial and continued surgical training. The lack of domestic simulators forces trainees to

either bear the cost of travel for extensive training or to operate on patients without the most recent techniques. Moreover, the current pandemic only exacerbates the cost and logistics of such endeavors. Therefore, access to surgical simulators serves an essential, yet under-discussed, role in the global health landscape.

Some solutions to this paucity of training modalities are surgical suture kits, which consist of a tri-layer silicone skin mold, sutures, and surgical instruments. In addition, knot tying trainers that include transparent knotting tubes, cylinders, and sutures are also available. However, such training platforms can cost up to $105 and $194 respectively. In many instances either this cost is prohibitive, manufacturers will not ship to the country in question, or it remains impossible to order or receive individual replacement components.

Drs. Yihan Lin, Emily Downs, Jason Han, and John Kelly initially designed the GlobalSurgBox. This trainer is assembled from common supplies that can be gathered in one’s home or local stores, and for that reason it costs only $30 per kit. The GlobalSurgBox is a surgical trainer that fits completely within a mobile 12.5-inch toolbox. The lid of the toolbox has several compartments which store sutures, needles, and other supplies. Inside the toolbox, there is a removable tray which contains necessary instruments required for practice such as a needle driver, pick up, and scissors. At the base of the toolbox, a wooden board with precisely placed nails serves as the foundation of all the surgical training exercises. All the materials needed for the creation of the box will be sourced from Amazon.com and Home Depot for ease of replication. In addition, locally available materials may be used to replenish supplies in parts of the world with less access to online commerce. The GlobalSurgBox allows for training of techniques such as square knot tying, tying knots “in a hole,” end-to-end vascular anastomosis, LIMA to LAD coronary anastomosis, and aortic valve replacement.

**Budget:** Use the GSSA google doc forms to locate the number of materials needed per toolbox and the price per item/set. Calculate how many boxes can be made with the grant/scholarship funding.

**Personal Statement:** Describe the importance of the GlobalSurgBox and its potential impact both locally and globally.

**LOR:** Ask Faculty Advisors for Letters of Recommendation, they are always willing to help!

4. Additional notes

- We have managed to optimize costs such that the cost price of each box is $25. However, when asking for funding, we challenge those using this document to have a “make one locally, give one globally” mindset. Therefore, we recommend $50 so 2 boxes can be made each time, with the second box to be sent ot a partner in an LMIC. If you are a program that needs to find partners who want these boxes, please reach out to us, we have many contacts available.

# GATHERING SUPPLIES

**Step 1: Determine the number of boxes you are planning to make**

To ensure that the cost price of each GlobalSurgBox is at its minimum ($25), we suggest making >20 boxes at a time.

**Step 2: Prepare your spreadsheet**

We have compiled a comprehensive list of all the materials we use to make the GlobalSurgBox. We have chosen materials from Amazon for maximum convenience. All of these materials have been vetted for quality as well as price. The list of items are on the below spreadsheet and also on an Amazon list. You can use this spreadsheet to calculate the number of items you need to buy, based on Step #1 (the number of boxes you've decided to make).

Please Copy our spreadsheet. **Do not edit the spreadsheet directly**.

https://docs.google.com/spreadsheets/d/1WZ5DYWo55uDEpaccqug4DqhBUEpeBa-2ze2t2uKET-w/copy?usp=sharing

**Step 2: Solicit for Surgical Supplies Donations**

Amend the poster found in Appendix 5: Request for Surgical Supplies to solicit donations of surgical instruments and suture. Amend wherever *** is noted with your organization, contact information and make modifications for which tools you are soliciting. Disseminate to the operating room, nurses stations, surgical interest groups, medical student liaisons, PAs/NPs offices, etc. Ensure enough time for collection prior to the box building event.

**Step 3: Purchase Specialty Tools**

If required purchase specialty tools. These may include castroviejos, special pick ups, etc. We have found the following company with decent quality tools at a reasonable price. Instructions are also in this spreadsheet:

https://docs.google.com/spreadsheets/d/1eEazG5vRtKsmUu3YWrL93knVgBoZFXMijtZC8wZagRo/edit?usp=sharing

**Step 4: Inventory**

1. Delegate someone responsible to count all items currently on hand and add to column H of the spreadsheet.
2. Compare Column H with column F to determine how many units are needed for each item.
3. Use column D to determine # of whole packages will be needed, the obtain # of units needed and add to column I.
4. Once total inventory is complete ensure the value in Cell L 34 is within your budget.

Example: Row 8: Alligator Clips. When inventoried 101 are available. 101 is placed in column H. Per Column F 300 total are needed. This means that 199 need to be ordered. As they are not ordered singly you look to column D. Per column D 100 are available in each package. As such you will need 2 whole packages in order to obtain 199 more. Put 2 in column I. This will automatically calculate the cost of your new order in column L.

**Step 5: Prepare your Order and Purchase**

1. We recommend ordering 1 month in advance.
2. Obtain the wooden boards from Home Depot, these are cut for free and may be cut into 3 pieces of 11 inches each. Be aware that this may require trips to multiple locations.
3. Be aware that toolboxes are often in short supply and these may need to be ordered in waves.

# Box Building Tips

1. Schedule a box building night with your team.
2. Order all required materials way in advance.
   a. Quality check the toolboxes prior to the event to make sure they are all in good condition (often a few are broken during transport)
3. The boxes should be built according to the instructions in this manuall titled: Set Up Instructions
4. The rate limiting step for building the GlobalSurgBoxes is the wooden boards. It takes time to buy the boards, get them cut, and hammering all the nails in - for efficiency, we suggest getting this done ahead of time and having all team members make these themselves, and bring them to the box building night for the additional steps.
5. During the box building night, set up an assembly line. One person should delegate jobs in the line to ensure that all items are included.
6. Bring scissors, hammers, and drills to the box building.
7. At the end of the night, quality check all of the completed boxes.

# Skills Night

1. Select date and time for training.
2. Reserve a room.
3. Solicit for volunteers to train students: from residents, fellows, attendings, senior students, etc.
4. Advertise: You may wish to use the poster template found in Appendix 4: Skills Night Poster. Amend the poster wherever a *** is noted with your information.
5. Arrive early to set up.
6. Introduce the GlobalSurgBox and its purpose.
7. Play the module training videos found at WWW.GLOBALSURGBOX.COM which are applicable to what you'd like to train that day.
8. Have Fun!

# GlobalSurgBox Setup Instructions

WWW.GLOBALSURGBOX.COM

**WHAT THIS SECTION IS ABOUT:**

The GlobalSurgBox is a personal, portable, and affordable surgical simulator.
We have put together instructions on how to make a GlobalSurgBox so you can make one for yourself, and also make one for a partner in a low- or middle-income country.

The GlobalSurgBox has three major components: The base of the toolbox, the middle removable tray, and the lid. All items can be found on Amazon or local home improvement stores. We have created an Amazon list of all our preferred materials that you can refer to - these have been vetted for quality and price. We have also created a pricing excel sheet if you are planning to make these in bulk and want to calculate quantities to purchase.

This document was created by the University of Colorado Global Surgery Student Alliance (GSSA) with mentorship from Dr. Yihan Lin. For any questions or suggestions, please email us at: globalsurgbox@gmail.com, cugssa@gmail.com and yihan.lin@gmail.com.

This portion of the document was last updated on 2022.03.26.

**COMPARTMENT 1: THE TOOLBOX BASE**

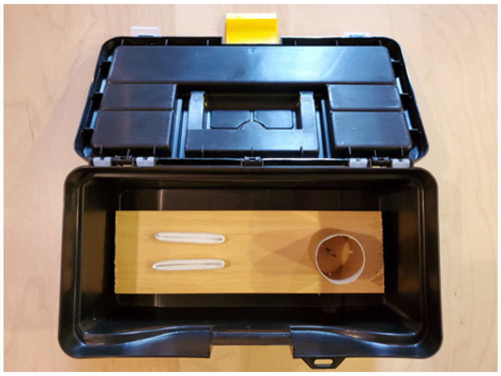

**Items:**

- 12-inch toolbox (1)
- Wooden board (1)
- Nails (8)
- Screw Eye Hook (1)
- Sturdy Cardboard Tube (1)
- Furniture Bumpers (4)
- Velcro Strips (2)

**Instructions:**

1. Wooden board
    a. The final dimension of each wooden board should be ~27.5 x 8cm.
    b. *Note: We purchase long wooden boards from Home Depot, please see our supplies list. Each long board can be cut into 3 pieces to fit into the toolbox. Ask assistance from Home Depot, as they have an automatic saw to help with this (for free). We highly recommend using the specific type/model of board in our supplies list. Boards that are a harder/thicker material are difficult to hammer nails into. Also, boards that are of lower quality will splinter easily, and are not ideal.*
2. Hammer Nails (8) and screw hook (1) to the wooden board (8) to match the following design.
    a. 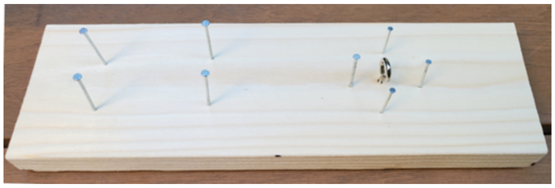 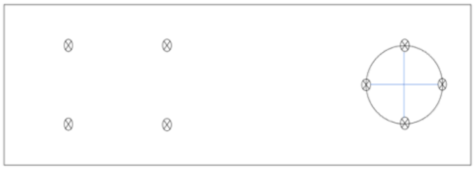
    b. *Note: The 4 nails on the right side of the board are meant to hold a round cardboard tube tightly in place. Therefore, precise placement is important as if it is too far apart, the cardboard tube will not fit above it, but if it is too loose, the cardboard tube will not be secure.*
    c. *Appendix 2: Real-size templates for wooden boards is a real life size template that you can print for use on the actual board.*
    d. *As you design more modules, please feel free to create your own designs for nail placement.*
3. Sturdy cardboard tube
    a. Cut to 1 inch segment and place over the 4 nails on the right side of the board
    b. *Note: This is used to practice "tying in a hole". We recommend using a hard cardboard tube (see our supplies list) for durability. Alternatively, you can use a toilet paper roll.*
4. Attach furniture bumpers to the bottom corners of the board

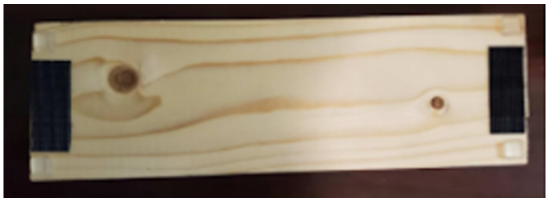 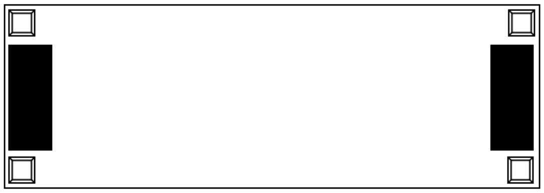

    a. Furniture bumpers: 4 furniture bumpers to be placed in the 4 corners on the bottom side of the board. We recommend using bumpers that are >3mm thick (has to be thicker than the velcro strip to work well).
    b. *Note: Furniture bumpers are used to prevent the board from sliding on the table while you practice.*
5. Attach two velcro strips to the bottom of the board
    a. Velcro strips: Cut two pieces into 5cm x 1cm segments. The velcro strips have two sides to them (thin/thick). Attach the two sides of the velcro together. Take off the sticky tape on the thinner side of the velcro and attach to the board. Then remove the second sticky side of the velcro and place the entire board in the base of the toolbox.
    b. *Note: The velcro strips are for advanced practice - once you feel comfortable with your skills, place the board inside the toolbox (velcro strips secure the board so it doesn't move in the toolbox), and now practice working "in a body cavity".*

**COMPARTMENT 2: THE REMOVABLE TRAY**

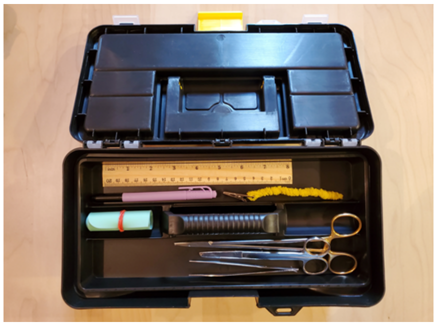

**Items:**

- Surgical Instruments: Needle driver, Pickups, Scissors
- Baking sheet (1)
- Yoga Pad (1)
- Gear tie attached to alligator clip
- Optional: Ruler 6-12 inches, marking pen, description card

**Instructions:**

1. Surgical instruments
    a. Minimum requirements: needle driver, pickups, scissors. For advanced trainees: castroviejo needle driver for vascular anastomosis practice.
    b. *Note: We get most of our instruments for free (the operating room discards tons of instruments that are non-functional for surgery but great for simulation practice). Alternatively, we have some options on our Amazon list you can purchase. For Univ of Colorado users, see our document on "Where to collect surgical tools at UCH" for all our collection centers around the hospital.*
2. Baking sheet
    a. Please see Amazon list, we recommend 0.02-0.05mm thickness. Each baking sheet can be cut up into several pieces. We roll up our baking sheets and keep it compact/neat with a rubber band.
    *b. Note: These are used for suturing practice (interrupted, running, vertical/horizontal mattress, etc)*
3. Yoga pad

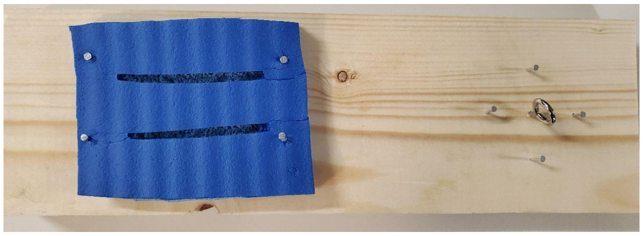

    a. Cut yoga mat into 10 cm x 8 cm sections. This should fit over the 4 nails of the wooden board to hold it in place. Cut partial thickness slits in the yoga pad using a scalpel/razor.
    *b. Note: These are used for subcuticular suturing practice.*
4. Gear tie attached to alligator clip
    a. Cut gear tie to around 10 inches. Twist into end of alligator clip.
    b.

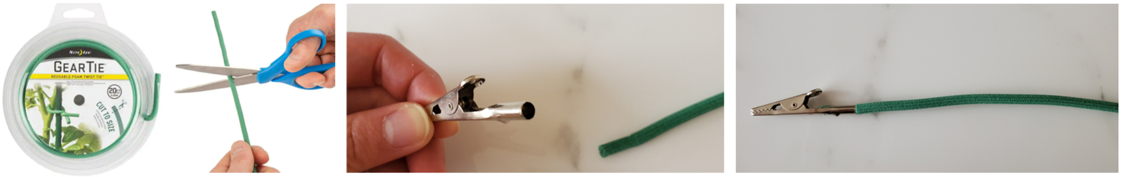


    *c. Note: This is used for vascular anastomosis modules to hold up the linear balloons, such that modules can be done without an assistant.*
    *d. Note: Alternatives to this include Nite Ize 6 inch gear ties (more expensive but very sturdy), or pipe clears (cheaper but much less sturdy, we recommend twisting several together if you use this).*
        *i.*

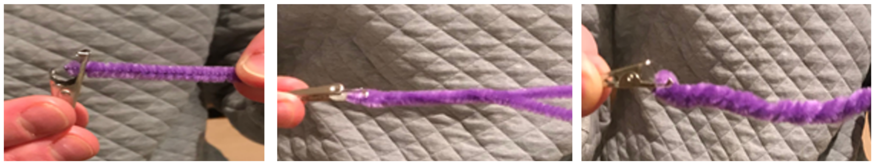

5. Description card
    a. Print Appendix 3 in color, double-sided. For Univ of Colorado users, download the file from the shared Google Drive.
    b. Use the paper cutter to cut out each card as thin as possible (minimize white spaces).
    c. Put 4 cards into one laminating sheet and laminate (turn laminator on in advance for it to warm up)
    d. Use the paper cutter to cut out each card, minimizing clear space so it fits in the toolbox tray.

**COMPARTMENT 3: THE LID**

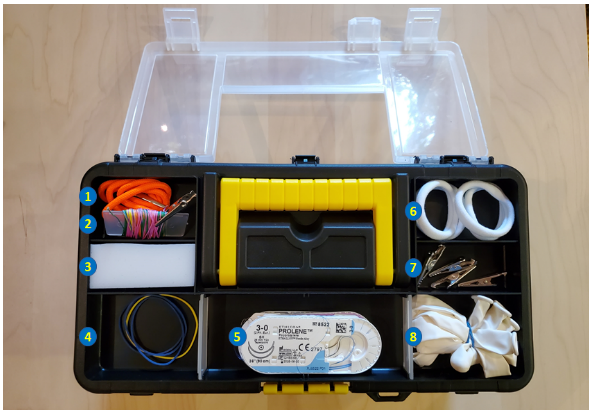


**Items:**
- 1) Shoelace
- 2) Braided fishing line
- 3) Sponge
- 4) Rubberbands
- 5) Sutures
- 6) Hair ties (4)
- 7) Alligator clips (4)
- 8) Linear balloons (5)
- Laminated Name Tag (1)

**Instructions:**
1. Cut the full length shoelace in ½, make a knot on the cut end, secure with an alligator clip.
2. Take braided fishing line and wrap it around one of the toolbox lid dividers, secure with an alligator clip.
    a. *Note: shoelace and fishing line are used for basic and advanced knot tying.*
3. Cut sponge so it fits into one of the lid compartments.
    a. *Note: This holds all your used needles so you don't get stuck*
4. Rubberbands: miscellaneous use
5. Sutures: Find for free at your operating room, or buy on Amazon.
6. Hair ties: used for suturing practice
7. Alligator clips: used to secure your skills station onto the nails on the board.
8. Linear balloons: used for vascular or bowel anastomosis practice.
9. Laminated name tag
    a. 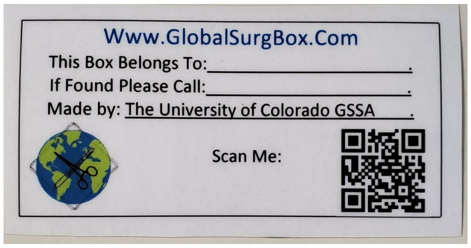

    b. Place these above sutures in the middle lid compartment for maximum visibility.
    c. To make a laminated name tag, please see Appendix 2 - print, laminate and cut.

Supplement to: Lin Y, Han JJ, Kelly JJ, Gergen AK, Downs E. Development of a modular and equitable surgical simulator. Glob Health Sci Pract. 2022;10(3):e2100744. https://doi.org/10.9745/GHSP-D-21-00744



Appendix 1: Real-size templates for wooden boards

**TOP**

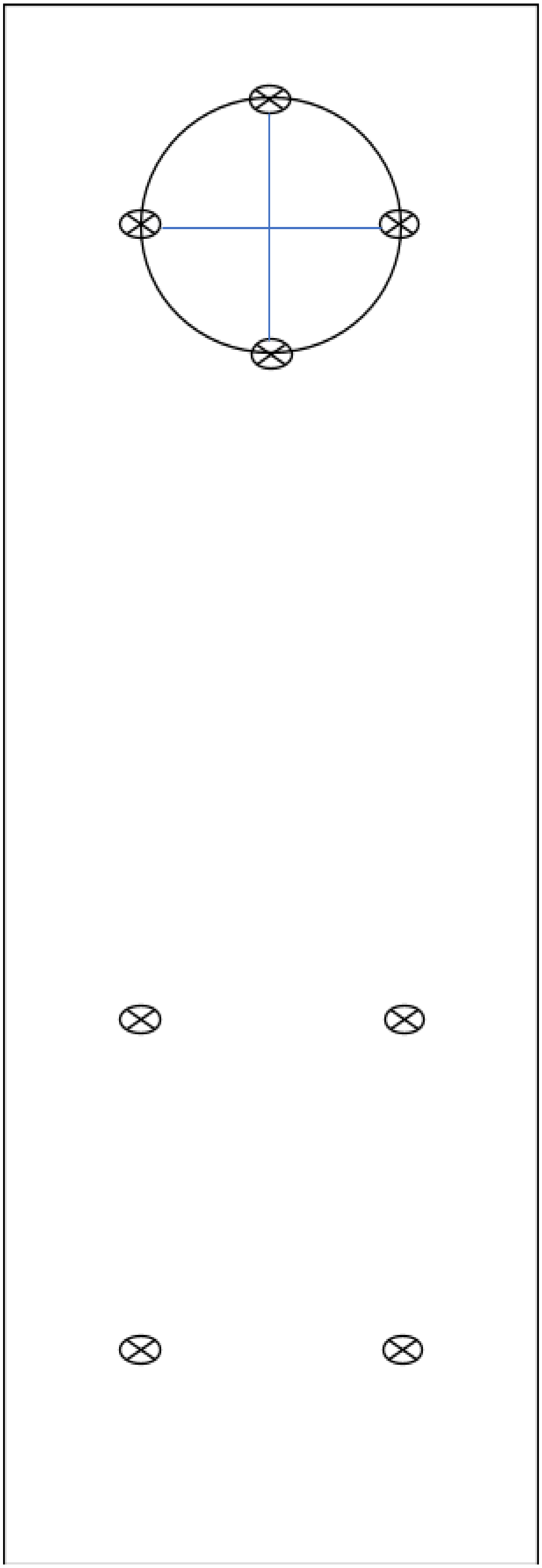

**Board: 27.5 X 8.5 CM**

**Top**

4cm X 4cm circle in the middle of the board ~3 cm from top, draw a cross, place eye hook at the line intersections

4 Nails total at 16 and 24 cm down from top AND at 2.75 and 5.75 cm in from left

**Base**

4 furniture bumpers placed at each corner

2 halves of Velcro pads (~5cm length) placed between bumpers as shown

**BASE**

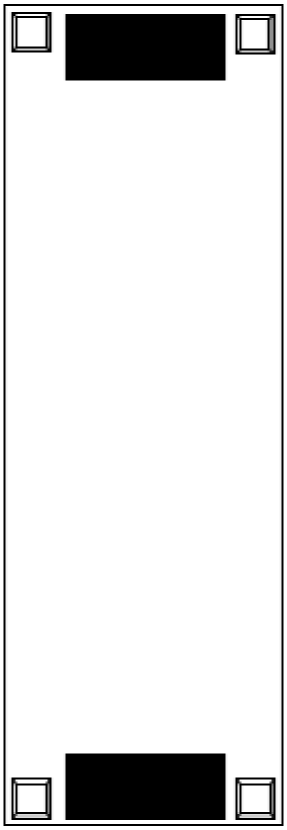

Supplement to: Lin Y, Han JJ, Kelly JJ, Gergen AK, Downs E. Development of a modular and equitable surgical simulator. Glob Health Sci Pract. 2022;10(3):e2100744. https://doi.org/10.9745/GHSP-D-21-00744



Appendix 2: Laminated Name Tags

| | |
|---|---|
| www.**GlobalSurgBox**.com<br>This Box Belongs To:________________.<br>If Found Please Call:________________.<br>Made by: The University of Colorado GSSA .<br>Scan Me: | www.**GlobalSurgBox**.com<br>This Box Belongs To:________________.<br>If Found Please Call:________________.<br>Made by: The University of Colorado GSSA .<br>Scan Me: |
| www.**GlobalSurgBox**.com<br>This Box Belongs To:________________.<br>If Found Please Call:________________.<br>Made by: The University of Colorado GSSA .<br>Scan Me: | www.**GlobalSurgBox**.com<br>This Box Belongs To:________________.<br>If Found Please Call:________________.<br>Made by: The University of Colorado GSSA .<br>Scan Me: |
| www.**GlobalSurgBox**.com<br>This Box Belongs To:________________.<br>If Found Please Call:________________.<br>Made by: The University of Colorado GSSA .<br>Scan Me: | www.**GlobalSurgBox**.com<br>This Box Belongs To:________________.<br>If Found Please Call:________________.<br>Made by: The University of Colorado GSSA .<br>Scan Me: |
| www.**GlobalSurgBox**.com<br>This Box Belongs To:________________.<br>If Found Please Call:________________.<br>Made by: The University of Colorado GSSA .<br>Scan Me: | www.**GlobalSurgBox**.com<br>This Box Belongs To:________________.<br>If Found Please Call:________________.<br>Made by: The University of Colorado GSSA .<br>Scan Me: |
| www.**GlobalSurgBox**.com<br>This Box Belongs To:________________.<br>If Found Please Call:________________.<br>Made by: The University of Colorado GSSA .<br>Scan Me: | www.**GlobalSurgBox**.com<br>This Box Belongs To:________________.<br>If Found Please Call:________________.<br>Made by: The University of Colorado GSSA .<br>Scan Me: |
| www.**GlobalSurgBox**.com<br>This Box Belongs To:________________.<br>If Found Please Call:________________.<br>Made by: The University of Colorado GSSA .<br>Scan Me: | www.**GlobalSurgBox**.com<br>This Box Belongs To:________________.<br>If Found Please Call:________________.<br>Made by: The University of Colorado GSSA .<br>Scan Me: |

Supplement to: Lin Y, Han JJ, Kelly JJ, Gergen AK, Downs E. Development of a modular and equitable surgical simulator. Glob Health Sci Pract. 2022;10(3):e2100744. https://doi.org/10.9745/GHSP-D-21-00744



# Appendix 3: Description Cards

**Welcome to the GlobalSurgBox!** Here is a map of all the items in the GSB.

*Need additional supplies? Visit: www.**GlobalSurgBox**.com for links on these materials and alternatives!*

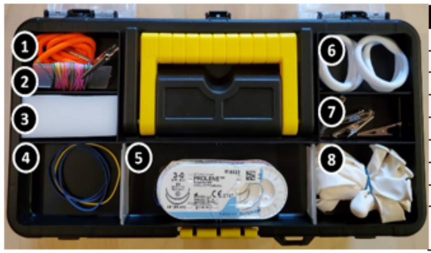


| IN THE LID | | |
|---|---|---|
| # | *Item* | *Purpose* |
| 1 | Shoelace | Knot tying (simple) |
| 2 | Fishing line | Knot tying (advanced) |
| 3 | Sponge | Store your needles |
| 4 | Rubber bands | Miscellaneous |
| 5 | Sutures | Suturing practice |
| 6 | Hair ties | Suturing practice |
| 7 | Alligator clips | Various uses |
| 8 | Linear balloons | Anastomosis: vascular, bowel |

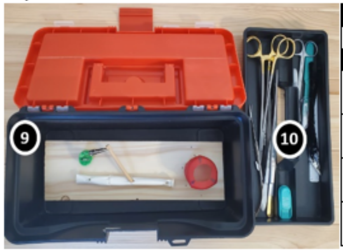


| IN THE MAIN COMPARTMENT | | |
|---|---|---|
| 9 | Board + nails | Use this to create any simulation module |
| **IN THE REMOVEABLE TRAY** | | |
| 10 | Surgical Instruments | Needle driver, Pick ups, Castroviejo (optional), Shod/snap (optional) |
| 11 | Baking sheet | Laceration repair, aortotomy closure, vascular patch, aortic cannulation |
| 12 | Cardboard cynlinder | "Tie in a hole", aortic valve replacement (alternative: toilet paper roll) |
| 13 | Pipe cleaners | Holding sutures, holding grafts/patches for vascular anastomosis |

## SAMPLE MODULES

*For additional modules, including accompanying instructional videos, please visit: www.**GlobalSurgBox**.com*

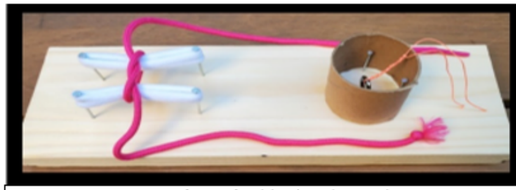

**Square knot tying** (shoelace, hair ties)
**Tying in hole** (Cardboard cylinder, fishing line)

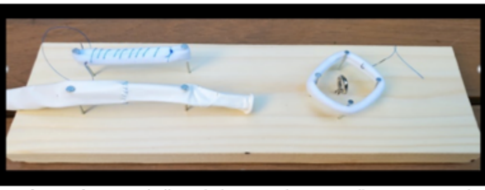

**Basic suturing** - Baseball stitch, horizontal mattress (hair tie, sutures)
**End to end vascular anastomosis** (linear balloon)
**Needle angle practice** (hair tie)

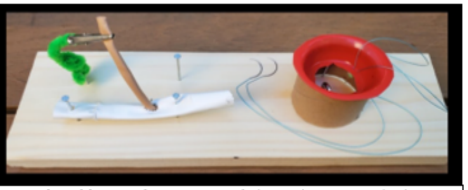

**End to side vascular anastomosis** (pipe cleaner attached to alligator clips, linear balloons)
**Aortic valve replacement** (cupcake silicone sheet, cardboard cylinder)

**Welcome to the GlobalSurgBox!** Here is a map of all the items in the GSB.

*Need additional supplies? Visit: www.**GlobalSurgBox**.com for links on these materials and alternatives!*

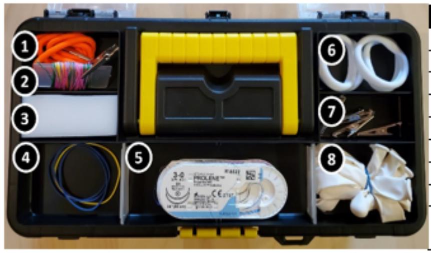


| IN THE LID | | |
|---|---|---|
| # | *Item* | *Purpose* |
| 1 | Shoelace | Knot tying (simple) |
| 2 | Fishing line | Knot tying (advanced) |
| 3 | Sponge | Store your needles |
| 4 | Rubber bands | Miscellaneous |
| 5 | Sutures | Suturing practice |
| 6 | Hair ties | Suturing practice |
| 7 | Alligator clips | Various uses |
| 8 | Linear balloons | Anastomosis: vascular, bowel |

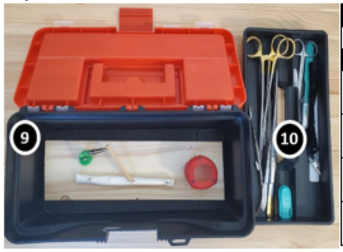


| IN THE MAIN COMPARTMENT | | |
|---|---|---|
| 9 | Board + nails | Use this to create any simulation module |
| **IN THE REMOVEABLE TRAY** | | |
| 10 | Surgical Instruments | Needle driver, Pick ups, Castroviejo (optional), Shod/snap (optional) |
| 11 | Baking sheet | Laceration repair, aortotomy closure, vascular patch, aortic cannulation |
| 12 | Cardboard cynlinder | "Tie in a hole", aortic valve replacement (alternative: toilet paper roll) |
| 13 | Pipe cleaners | Holding sutures, holding grafts/patches for vascular anastomosis |

## SAMPLE MODULES

*For additional modules, including accompanying instructional videos, please visit: www.**GlobalSurgBox**.com*

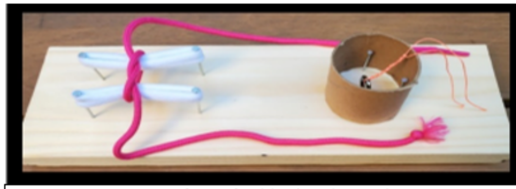

**Square knot tying** (shoelace, hair ties)
**Tying in hole** (Cardboard cylinder, fishing line)

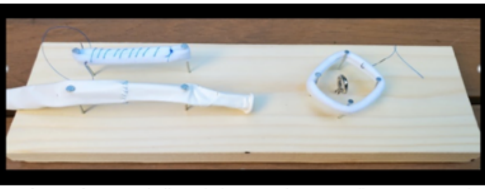

**Basic suturing** - Baseball stitch, horizontal mattress (hair tie, sutures)
**End to end vascular anastomosis** (linear balloon)
**Needle angle practice** (hair tie)

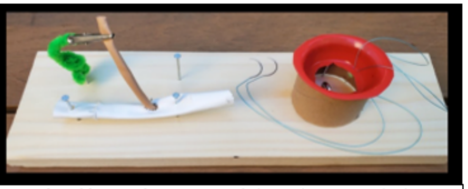

**End to side vascular anastomosis** (pipe cleaner attached to alligator clips, linear balloons)
**Aortic valve replacement** (cupcake silicone sheet, cardboard cylinder)

Appendix 4: Skills Night Poster

# IN PERSON SURGICAL SKILLS NIGHT!

****DAY, ***, 202*** ***-***PM**
**RSVP: *****

Come join the Global Surgery Student Alliance for our
SURGICAL SKILLS NIGHT!!!!

For this skills session, we will be utilizing a personal, portable, cost-effective surgical simulation kit - the GlobalSurgBox (www.globalsurgbox.com).
All participants will be able to receive this toolkit FREE at the end of the session.

Bonus: For each kit given locally, an additional simulation kit will be sent to our partners in a low- or middle-income country for residents and students to train!

*** will be training us on one-handed and two-handed knot tying as well as basic suturing utilizing the toolkit supplies. The event is currently limited to *** respondents so be sure to RSVP at the link ABOVE. The first *** will receive an email from me with more information. Hope to see you there!

Appendix 5: Request for Surgical Supplies

# REQUEST FOR SURGICAL SUPPLIES

**The Global Surgery Student Alliance (GSSA) at the ***-Medical School is looking for donations to supply a GlobalSurgBox initiative. We intend to use these instruments to create durable and affordable surgical simulations kits for our partners in low-resource settings. If you come across any of the below instruments, sutures, or ties, please consider donating them to the GSSA.**

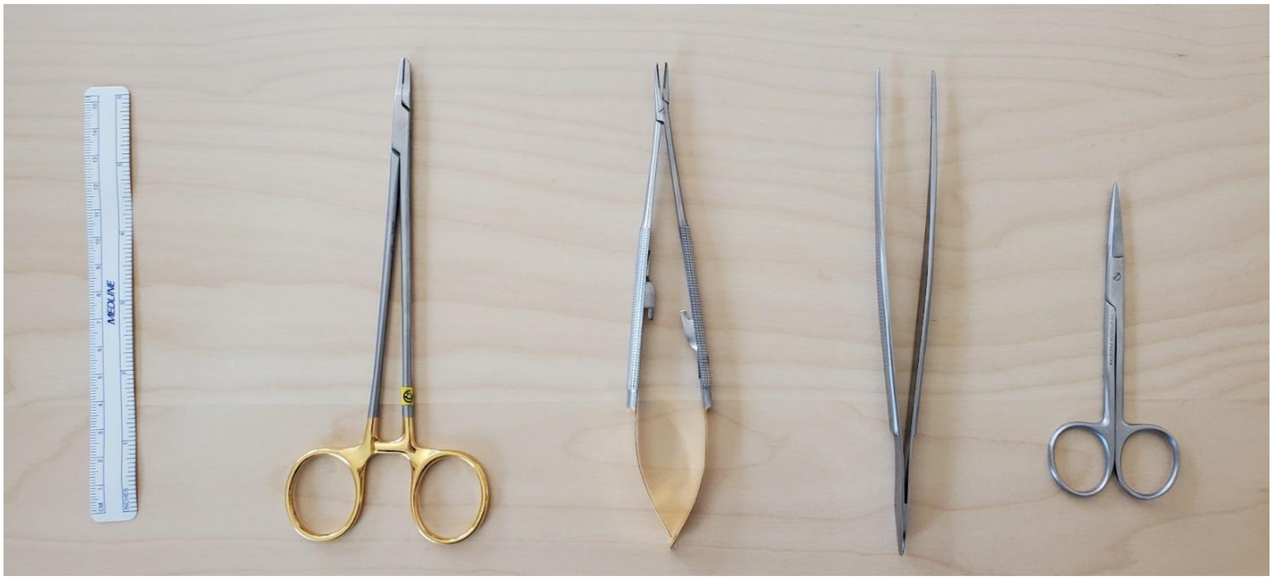


| Ruler | Needle Driver | Castroviejo Needle Holder | Forceps | Scissors |
|---|---|---|---|---|

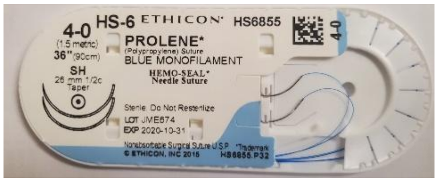


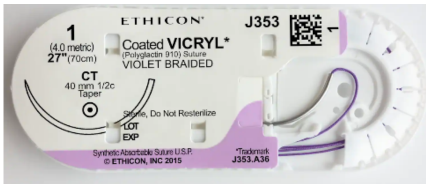


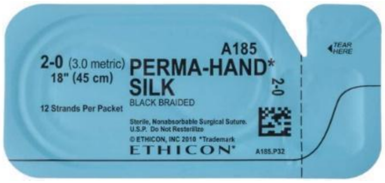


| Monofilament suture<br>*(any size)* | Braided suture<br>*(any size)* | Ties<br>*(any size)* |
|---|---|---|

**For donation pick up, please contact *** (***@***.***)**
**To learn more about the GlobalSurgBox initiative, please visit www.globalsurgbox.com.**
